# Lipid Microbubble–Conjugated Anti-CD3 and Anti-CD28 Antibodies (Microbubble-Based Human T Cell Activator) Offer Superior Long-Term Expansion of Human Naive T Cells In Vitro

**DOI:** 10.4049/immunohorizons.2000056

**Published:** 2020-08-07

**Authors:** Ana Lustig, Ty’Keemi Manor, Guixin Shi, Jiangyuan Li, Ying-Ting Wang, Yang An, Yu-Tsueng Liu, Nan-ping Weng

**Affiliations:** * Laboratory of Molecular Biology and Immunology, National Institute on Aging, National Institutes of Health, Baltimore, MD 21224; † Diagnologix LLC, San Diego, CA 92121; ‡ Laboratory of Behavioral Neuroscience, National Institute on Aging, National Institutes of Health, Baltimore, MD 21224; § University of California, San Diego, San Diego, CA 92093

## Abstract

Stimulation of human primary T cells with immobilized anti-CD3 and anti-CD28 Abs in vitro provide a system to study T cell activation and proliferation and an avenue for expanding T cells for immunotherapy. Magnetic beads conjugated with anti-CD3 and anti-CD28 Abs (Dynabeads Human T-Activator [D-TCA]) have been a golden standard for stimulating human primary T cells in vitro. In this study, we report that an application using anti-CD3 and anti-CD28 Abs conjugated on lipid microbubbles (microbubble-based human T cell activator [MB-TCA]) to stimulate primary human naive T cells resulted in expansion superior to D-TCA. In 56-d cultures with three repeated stimulation cycles (14 d per stimulation), we found that 1) MB-TCA induced significantly better expansion (20- and 10-fold increase) of naive CD4^+^ and CD8^+^ T cells than did D-TCA; 2) MB-TCA–and D-TCA–stimulated T cells had a similar number of initial cell divisions, but MB-TCA had significantly lower activation-induced cell death than D-TCA; 3) MB-TCA–stimulated T cells produced less TNF-a than did D-TCA; and 4) blocking TNF-a action via adding an Ab against TNF-αR (TNFRSF1A) significantly improved expansion of T cells activated by D-TCA in vitro. Together, we demonstrated that the MB-TCA induces a better expansion of human naive T cells in vitro and offers advantages in both basic and clinical applications in which the outcome depends on the number of T cells. *ImmunoHorizons*, 2020, 4: 475–484.

## INTRODUCTION

In an adaptive immune response, activation of naive T cells is initiated by an engagement between TCR and Ag presented by MHC on the surface of APCs, along with interactions between the costimulatory receptors, such as CD28 and its CD80/86 receptor (1). Activated T cells then undergo a series of steps involving proliferation and differentiation to become effector cells first and eventually to differentiate to memory T cells (2, 3). Thus, naive T cell activation and differentiation are required for eliminating the pathogen and establishing long-lasting immune protection. As T cell activation is carried out in an Ag-specific manner, study of the mechanisms of T cell activation is limited by the available number of T cells expressing any specific TCR.

The identification and characterization of mAbs that recognize TCR-CDR3 revealed the ability of an anti-CD3 mAb (OKT3) to induce DNA synthesis of resting human T cells in vitro (4). Subsequent studies demonstrated that naive T cells require stimulation not only of the TCR, but also additional signals such as a costimulatory signal from CD28 (5) and cytokines such as IL-2 (6, 7) to optimize the proliferation response. Initially, anti-CD3 Ab was immobilized on the cell culture plate to achieve good activation. Subsequently, conjugation of anti-CD3 and anti-CD28 Abs to magnetic beads (Dynabeads Human T-Activator [D-TCA]) has substantially improved the efficiency and reproducibility of T cell activation because the size of Dynabeads resembles that of T cells and is suited for repeated use to allow for long-term autocrine growth of human T cells in vitro (8).

Further attempts to improve the T cell activation method have yielded some interesting findings in the past decade, including immobilizing the Abs on other surfaces such as polystyrene plastic and biopolymer-coated magnetic beads (9). It appears that the soft materials used for conjugating anti-CD3 and CD28 Abs induce higher IL-2 production and better in vitro proliferation of human T cells compared with more rigid substrates (9). More closely mimicking natural APCs, a fluid lipid bilayer supported by mesoporous silica microrods is conjugated with anti-CD3 and anti-CD28 Abs plus supplements of IL-2. This system induces 2–10-times better expansion of T cells than does D-TCA (10). Although anti-CD3 and anti-CD28 conjugated with the lipid-based biomaterials offers better expansion, D-TCA as a commercial product with good reproducibility is still a widely used reagent for activating human T cells in vitro in both basic research and clinical applications (11).

During T cell activation, cytokines act as the third signal in addition to the TCR (anti-CD3), and costimulatory receptors such as CD28 also play an important role in T cell proliferation and expansion (12). Once activated, T cells produce various cytokines including IL-2, IFN-γ, and TNF-α to further augment T cell expansion in both autocrine and paracrine fashion (13, 14). Although these T cell–produced cytokines are critical for the initial proliferation, IFN-γ and TNF-α also have been found to induce apoptosis in stimulated T cells, as demonstrated by blocking their effects using neutralizing Abs (15). The impact of activated T cell–produced cytokines on the growth and survival of T cells in long-term culture has not been fully determined.

In this study, we report a study using anti-CD3 and CD28 Abs conjugated to lipid microbubbles (MBs) (MB-based human T cell activator [MB-TCA]) and compare the long-term growth of human naive CD4^+^ and CD8^+^ T cells in vitro with the standard D-TCA. We found that MB-TCA–stimulated naive CD4^+^ and CD8^+^ T cells have greater expansion and lower cell death in vitro than those activated with D-TCA. Furthermore, we found that D-TCA induced significantly higher TNF-α and other cytokines in stimulated T cells than MB-TCA and that blocking the action of TNF-α significantly improved expansion of stimulated T cells. MB-TCA is inexpensive and relatively simple to be produced; thus, it could be an activator of choice for long-term expansion of human T cells in basic and clinical applications.

## MATERIALS AND METHODS

### Generation of anti-CD3- and anti-CD28-conjugated MBs (MB-TCA)

The procedure of generating MB production was previously described (16, 17). Briefly, the lipid mixture of DSPC/PEG40 stearate/DSPE-PEG3400-mal (molar ratio 10:1:1) in chloroform were dried in a 7-ml borosilicate glass vial (20mg total lipid) under a nitrogen stream to form a thin lipid film. The lipid film was rehydrated in 5 ml of PBS for 10 min, vortexed vigorously for 30 s, and briefly sonicated for 1 min to suspend the lipid mixture inPBS. The headspace of the vial was filled with a naturally stable perfluorohexane (PFH)/air gas mixture at atmospheric pressure and room temperature, which was achieved by filling a 10-ml syringe with 1ml of liquid PFH and then being allowed for inverted standing for 5 min to establish equilibrium between PFH gas and air inside the syringe. After the PFH/air gas mixture was carefully injected into the vial, the open end of the vial was sealed with parafilm. MBs were produced by probe sonication (5 s/cycle, six cycles total) using a Misonix XL-2000 probe Sonicator. MBs were washed at least three times by centrifugation at 50 × g for 1 min with PBS to remove the lipid suspension, film fragments, and small-size MBs. The maleimide-activated MBs were resuspended in PBS at a concentration of 2 × 10^8^/ml.

The MB-TCA was prepared by two approaches: 1) one-step conjugation, also called direct conjugation, with which MBs were directly conjugated with primary anti-human CD3/anti-human CD28; and 2) two-step conjugation, also called indirect conjugation, with which MBs were conjugated with a linker first, and then by anti-human CD3/anti-human CD28. We have compared the effects of both directly and indirectly conjugated MB-TCA and found they were comparable in T cell expansion (data not shown). We used indirectly conjugated MB-TCA in our reported findings in this study. For the indirect conjugation, anti-mouse IgG (Fc fragment specific) was activated by Traut’s reagent, followed by purification using a Zeba Spin Desalting Column. Immediately after the purification, the thiolated anti-mouse IgG was then reacted with premade maleimide-activated MBs and further conjugated with anti-human CD3 and anti-CD28 Abs (same molar ratio). The immuno-MBs were washed three times after each reaction to remove unreacted Abs by centrifugation. Finally, MBs were resuspended in PBS at 2 × 10^8^/ml and stored at 4°C prior to use.

### Isolation and culture of naive CD4^+^ and CD8^+^ T cells

Blood samples were collected from 43 healthy human adults under an institutional review board–approved protocol. Supplemental Table I shows a list of all donors used for various analyses. Naive CD4^+^ and CD8^+^ T cells were isolated from blood by negative immunomagnetic separation as previously described (18, 19). Briefly, PBMCs were isolated from whole blood using Ficoll (Ficoll Paque Plus, GE 17–1440-03; VWR Scientific, Philadelphia, PA) density centrifugation. The cells were washed and divided into two aliquots depending on expected total yield. Naive CD4^+^ and CD8^+^ T cells were isolated by the addition of Ab mixtures comprising Abs to CD11b (clone NIH11b-1), CD14 (63D3), CD16 (3G8), CD19 (FMC63), RBCs (10F7), MHC class II (L243) platelets (37F9-E7CD45RO [UCHL-1]), and either CD4 (O516) or CD8 (B9.8) for negative selection of naive CD4^+^ or CD8^+^ T cells. After 30 min of rotating in a 15- or 50-ml tube at 4°, the cells were washed and mixed with an equal volume of BioMag goat anti-mouse IgG magnetic beads (QIAGEN, Germantown, MD) and once again rotatedfor30minat4°.The cell/beadmixture is then placed into a magnet (Life Technologies, Carlsbad, CA), and the supernatant contains enriched naive CD4^+^ or CD8^+^ T cells.

Five million freshly isolated naive CD4^+^ and CD8^+^ T cells were mixed with an equal number of either Dynabeads Human T-act CD3/CD28 (Thermo Fisher Scientific, Waltham MA) or anti-CD3–and anti-CD28–conjugated MBs (MB-TCA) in 10 ml of RPMI 1640 supplemented with 10% FBS and an antibiotic solution containing L-glutamine (0.3 mg/ml), penicillin (50 U/ml), and streptomycin (50 μg/ml) (Thermo Fisher Scientific). The cell suspensions were then placed into 15-ml tubes on a rotator (Thermo Fisher Scientific) at 37°C for 30min. After that, the cells and beads (5mlvol) were placed into T25 culture flasks (Thermo Fisher Scientific) in an incubator with 37°C and 5%CO_2_.Depending on the growth of each culture, 5 ml of fresh culture medium was added within 3–5d (with an average of 5d). After 7–10d (with an average of 9d), cells were counted, and 10 million cells were reseeded in 15ml of culture medium containing 5ml existing culture medium plus 10ml of fresh medium transferred into a T75 flask. Every 14 d after the initial activation, the cells were counted, and 1–5 million cells were taken for subsequent analyses, including cell phenotype by flow cytometry, telomere length, and telomerase activity. Ten million cells from each culture were restimulated with the appropriate activation beads, rotated again as before, and resuspended in 20 ml of culture medium containing 10ml of the existing medium and 10ml of fresh medium transferred into a T75 flask. Typically, CD4^+^ T cells underwent four to five repeated stimulations, whereas CD8^+^ T cells underwent three to four stimulations before termination of the culture when the majority of the cells were dead or dying.

To determine if MB treatment of cells previously activated with Dynal beads could restore the cell growth levels to that of cells previously activated with MBs, similar cultures were prepared. The cultures were activated on day 0 and then divided on day 10 into two cultures each, which were reactivated with each of the bead types. The cells were counted on day 20.

### Proliferation analysis based on cell counts

At each time point, cells were suspended in trypan blue solution and counted either using a hemocytometer or a Cellometer (Nexcelom Bioscience, Lawrence, MA). Proliferation was calculated as the fold increase based on current cell counts divided by the seeded cell counts at each time point, and the overall fold change was calculated as the current cell count divided by the count at the previous time point, then multiplying that number by the fold change at the previous time point.

### Cell division analysis using CFSE

Two million cells were incubated in 1 ml of Dulbecco’s PBS (Mediatech, Manassas, VA) with 5 nM CFSE (BioLegend, San Diego, CA) for 10 min at 37°. The cells were then washed twice with 5 ml of culture medium, divided into two, activated as described above with either D-TCA or MB-TCA, and put into culture. Four days after the first and the second activation, each culture was counted, and CFSE intensity was measured on an FACSCanto II (BD Biosciences, San Jose, CA). The gating strategy used was lymphocytes (forward light scatter area [FSC-A] × side light scatter area [SSC-A]) and then singlets (FSC-A × forward light scatter height [FSC-H]) before focusing on CFSE. Proliferation comparison was done with Microsoft Excel and Modfit software (Verity Software House, Topsham, ME).

### Viability analysis by flow cytometry

One million cells from each culture were kept in the dark for 30 min at 4° with Ghost Dye Violet 510 (Tonbo Biosciences, San Diego, CA). The cells were washed in FACS buffer, fixed in PBS containing 2% paraformaldehyde, and analyzed on a BD FACS-CantoII (Becton Dickinson, San Jose, CA). The data collected were analysed using Flow Jo software (FlowJo, Ashland, OR).The gating strategy used was lymphocytes (FSC-A × SSC-A) and then singlets (FSC-A × FSC-H) before focusing on viability. For measurement of apoptosis, 1 million cells from each time point were incubated in the dark in annexin binding buffer with 5 μl each of annexin V–FITC and 7–aminoactinomycin D (7-AAD) (both from BioLegend) for 10 min at room temperature. A total of 0.5 ml of annexin binding buffer was then added to each culture and immediately analysed on an Accuri C6 cytometer (BD Biosciences). The gating strategy was as described above.

### Measurement of telomere length by fluorescent in situ hybridization with flow cytometry

Telomere length was measured by fluorescent in situ hybridization with flow cytometry and completed using a previously described protocol (20). In brief, 2 million cells from long-term cultures, and isolated PBMCs run analogously to serve as controls, were labelled with CD3(BV510) and farred fluorescent reactive dye (viability dye) and resuspended in FACS buffer (HBSS [Mediatech, Tewksbury, MA] with 1% BSA and 0.1% sodium azide). Abs were purchased from either BioLegend or Thermo Fisher Scientific. Each sample was divided equally into two Eppendor ftubes, one for telomere peptide nucleic acid fluorescent-probe “Tel Cy3” (F1002–5; Panagene, Daegeon, South Korea) and one without probe after cross-linking with bis (sulfosuccinimidyl) suberate (Thermo Fisher Scientific). The bis (sulfosuccinimidyl) suberate cross-linked samples were subjected to heat denaturation of DNA for 15 min at 87°C in a Thermomixer R (Marshal Scientific), followed by hybridization overnight at room temperature in the dark.

Cells were washed in FACS buffer and fixed in PBS containing 2% paraformaldehyde before being transferred to Falcon 2058 tubes (Becton Dickinson) and analyzed on a BD FACSAria Fusion (Becton Dickinson). The data collected were analyzed using FlowJo software (FlowJo). Gating schemes included total lymphocytes (SSC-AbyFSC-A), single cells (FSC-HbyFSC-A), viability (Viability-APC by CD3-Brilliant Violet 510) and Tel Cy3 mean fluorescence intensity (MFI) (count by Tel Cy3). The MFI of the last gate was calculated from difference between samples with probe and without probe and normalized against bovine lymphocyte controls used in each experiment. To convert MFI to quantifiable kilobase length, a conversion of the quantity of telomere fluorescence to actual telomere-terminal fragment length was conducted using a Southern comparative analysis from donors (*n* = 31) from weekly blood packs.

### Measurement of telomerase activity by TNF-related activation protein assay

Telomerase activity was measured as previously described (21). In brief, 0.5×10^6^ cellswere collected for each sample and lysed at 1× 10^6^cells/100 μl in CHAPS lysis solution supplemented with 0.1mM PMSF. Ten thousand cell equivalents of CHAPS lysate were used for telomere synthesis in a 10-μl volume containing the following components: 1× telomerase reaction buffer (50 mM Tris-OAc [pH 8.5], 1 mM MgCl_2_, 50 mM KOAc, 5 mM 2-ME, and 10 mM spermidine), 2 mM telomere dNTP mixture (dATP, dGTP, dTTP), and 1 mM of PAGE-purified Ts oligonucleotide. Two microliters of telomere synthesis solution were used in 15-μl PCRs, including 1.2 U of Platinum Taq, 1.5 μM MgCl_2_, 0.2 μM dNTP, 200 μM TAMRA-TS, NT, and ACX primers, and 0.1–1-amole TSNT oligonucleotide. PCR cycling conditions were as follows: 94°C for 2 min, followed by 32–35 cycles of 94°C 15 s and 60°C for 20 s. The PCR products were separated on nondenaturing 12% PAGE gels and fluorescent TAMRA-TS–amplified telomerase products were detected in the Cy3 channel on a Typhoon FLA7000 imaging system (GE Healthcare Life Sciences). Telomerase activity was quantified in ImageQuant or ImageJ as the ratio of the first six telomerase products to the TSNT internal control.

### Measurement of cytokines in activated cells and culture supernatants

CD4^+^ T cells were cultured with either D-TCA or MB-TCA as described. An aliquot of culture supernatant was saved on days 4, 10, 14, and 21 after the initial activation of the cells. Cytokine analysis was performed using a LEGENDplex Human Th1/Th2 Panel (eight-plex) analysis kit (BioLegend). Twenty-five microliters from each supernatant sample was analyzed for concentrations of IL-2, IL-4, IL-5, IL-6, IL-10, IL-13, IFN-γ (IFNG), and TNF-a (TNFa). Briefly, the samples were mixed with beads of various sizes coupled to Abs for each cytokine, followed by incubation with biotin-conjugated Abs and streptavidin–PE for detection. The results were measured using the Cytoflex flow cytometer (Beckman Coulter, Indianapolis, IN) compared against a standard curve provided in the kit, and the data were analyzed with LEGENDplex Data Analysis Software and Microsoft Excel. The cells were also directly measured by flow cytometry for percentage and expression levels of IFN-γ (BioLegend) and TNF-α (BioLegend) on a BD FACSCanto flow cytometer (Becton Dickinson).

### Blocking of cytokine receptors in culture

To determine the effects of blocking cytokine signaling pathways on activation and growth response of cultured lymphocytes, their corresponding receptors were blocked using anti-TNF(TNF-αRI/TNFRSF1A), anti-IFNG (IFN-γR1/CD119), and anti–IL-10Rα, (all from R&D Systems, Minneapolis, MN), either individually or in combination. Isolated naive CD4^+^ T cells were put into culture with D-TCA as described above and activated on day 0. Separate aliquots of the cells were activated as usual with D-TCA and cultured with each Ab or a combination of all three, at concentrations recommended by the company to inhibit in vitro cell growth by 50%. The cultures were counted on day 7, and the counts were again used as a measure of cell response and growth.

### Statistical analysis

The comparisons between the responses to the different modes of activation at each point by paired or unpaired Student *t* test. The differences of overall changes over multipoint time points between two stimulators were first computed by subtracting D-TCA from MB-TCA(MB-TCA–D-TCA) for each donor at each occasion. The objective is to test if the average difference between two conditions are significantly different from zero after predefined day. Because the measurements on the different days are clustered with in each donor. we took multilevel modeling approach. Linear mixed effects (LME) model was used with the difference as the outcome. Fixed effect and random effect are the intercept. Each donoris considered as a cluster. By computing the difference first and then use LME models, we account for the fact that the same donor is under two different conditions, and repeated measurements were taken on the same donor. Gehan–Breslow–Wilcoxon test was used for comparison the length of T cells in culture between D-TCA from MB-TCA stimulations.

## RESULTS

### Better expansion and viability of naive CD4^+^ and CD8^+^ T cells in response to MB-TCA than to D-TCA

**FIGURE 1.**
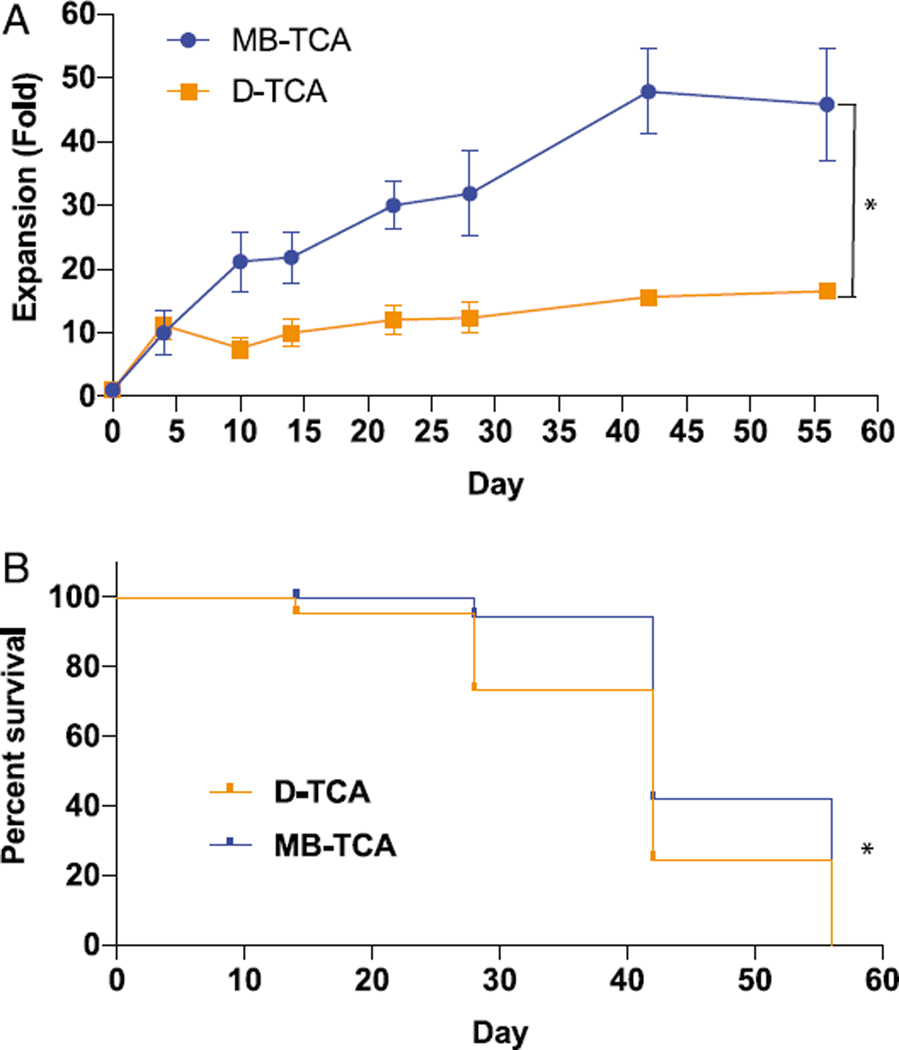
Expansion and longevity of activated naive CD4^+^ T cells in vitro stimulated by D-TCA and MB-TCA. **(A)** Naive CD4^+^ T cells were isolated from healthy human adults and activated at day 0 with either D-TCA or MB-TCA and restimulated every 14 d for the duration of the culture. At the designated time points, cells were counted, and expansion of cells was calculated as fold increase from the beginning of the culture. *p < 0.05 using the LME model, which was also used to compare the overall differences between the two activation responses. (**B**) Longevity of naive CD4^+^ T cells in culture stimulated by either D-TCA or MB-TCA. With naive CD4^+^ T cells from a total of eight subjects, four subjects stopped culture before day 42 when stimulated by D-TCA, whereas only one subject stopped before day 42 when stimulated by MB-TCA. *p < 0.05 using Gehan–Breslow–Wilcoxon test, which was also used for comparison of the overall differences between the two activation responses.

Human naive CD4^+^ and CD8^+^ T cells (CD45RA^+^CD62L^+^) were isolated from peripheral blood of healthy adults and stimulated with MB-TCA or D-TCA. Cell expansion in vitro was analyzed by cell counter at the designated days and restimulated every 14 d after each stimulation and presented as the fold change over the initial seeded cells. MB-TCA induced better expansion of both naïve CD4^+^ and CD8^+^ T cells than D-TCA did (Fig.1A,Supplemental Fig. 1D). This difference was observed as early as day 8 after stimulation and maintained up to 56 d in culture for naive CD4^+^ cells and 28 d in culture for naive CD8^+^ cells. Furthermore, fewer donors had T cells that survived to day 42 or longer with D-TCA (four of eight subjects, or50%) than with MB-TCA (seven of eight subjects, or 88%) (Fig. 1B). Similar findings were also observed in naive CD8^+^ T cell cultures (40 and 100% cultures reached day 28 under D-TCA and MB-TCA, respectively) (Supplemental Fig. 1E). To rule out whether the observed difference of these two stimulators was due to the different actual quantities of anti-CD3/CD28 molecules on the beads, we compared 1× and 3× concentration of both stimulators and found no substantial differences in cell expansion between the two concentrations for both stimulators (Supplemental Fig. 2).

**FIGURE 2.**
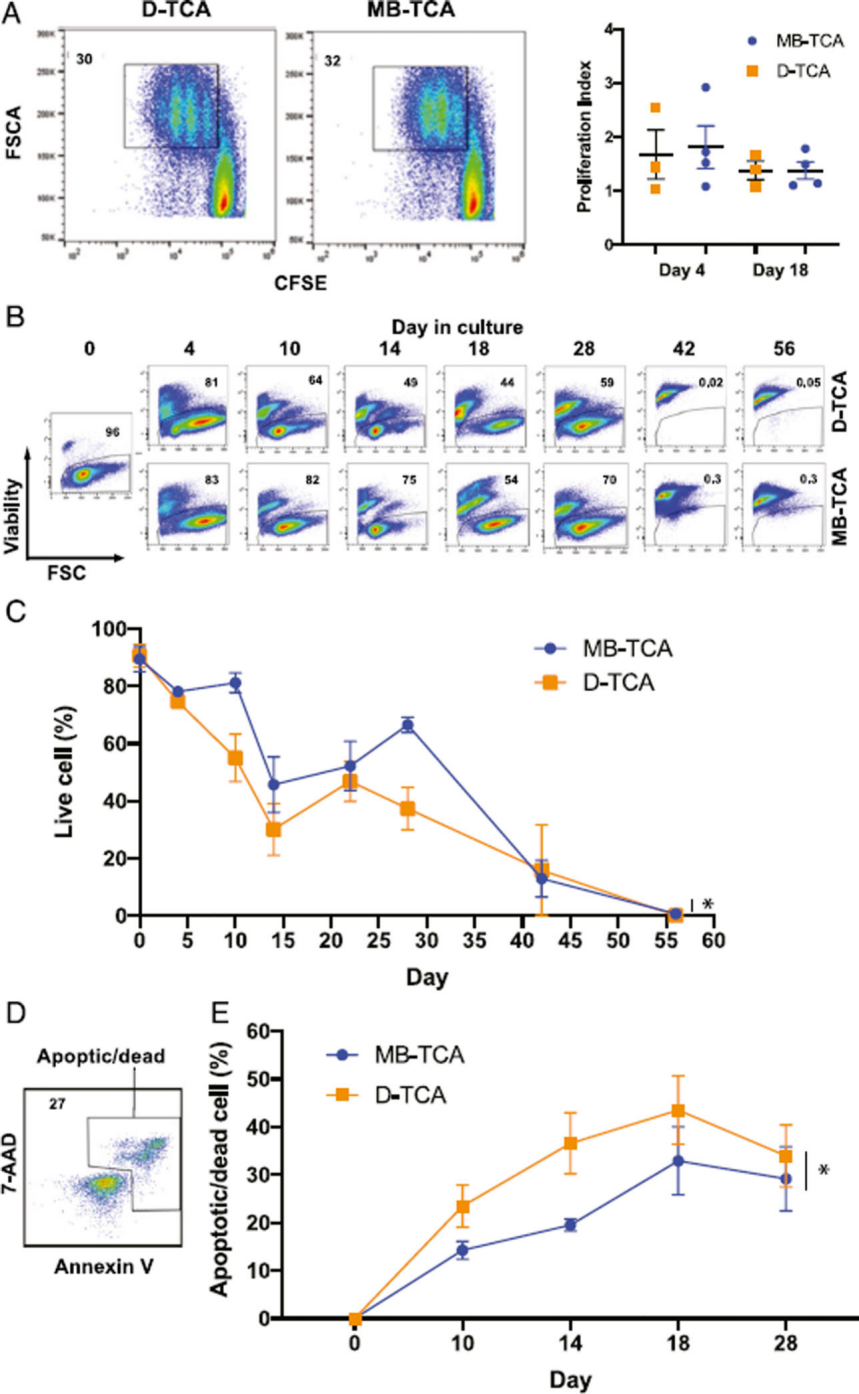
Cell division and death comparisons between D-TCA and MB-TCA. **(A)** Cell divisions after stimulation with either D-TCA or MB-TCA. Freshly isolated naive CD4^+^ T cells were labeled with CFSE and activated with either D-TCA or MB-TCA for 4 d, and data were collected by flow cytometry. Cell proliferation was determined by the proliferation index using Modfit software. There was no statistical difference between either mode of activation at day 4 after first stimulation or at day 18, 4 d after the second stimulation on day 14. **(B)** Representative graphs showing the dead cell dye exclusion by viable cells during the course of the cultures from one donor. Gating strategy was based on live cells on FSC-A and SSC-A and then gated on single cells. **(C)** Live cell percentages during 56-d culture. The average percent viable at each time of analysis was presented for both D-TCA– and MB-TCA–stimulated naive CD4^+^ T cells (*n* = 12). p = 0.04 using LME model, which was also used to compare the differences. **(D)** Representative graph of annexin V and 7-AAD staining. **(E)** Percentages of apoptotic/dead cells after the first 28 d of culture. The average of four experiments is shown. *p < 0.05 using Student paired t test and for all time points for comparison.

To determine mechanisms underlying the better expansion of naive CD4^+^ and CD8^+^ T cells stimulated by MB-TCA, we compared cell division and cell death between these two stimulators. Using CFSE tracking dye, we found a similar number of cell divisions between MB-TCA and D-TCA in naive CD4^+^ T cells at 4 d after the first stimulation and 4 d after the second stimulation (Fig. 2A). We then measured cell viability using the ability of cells to exclude a dead cell dye. We applied the LME model to compare the overall differences and found that naïve CD4^+^ T cells stimulated with MB-TCA had a significantly higher percentage of viable cells than those activated with D-TCA through out the course of culture (*p* =0.04) (Fig. 2B, 2C). To further determine whether the cell death was due to apoptosis, we used annexin V and 7-AAD staining and found that naive CD4^+^ T cells stimulated by D-TCA had a significant higher percentage of apoptotic and dead cells throughout the duration of the cultures when the expansion difference was observed (Student paired t test for all time points was used for comparison, and *p* = 0.02) (Fig. 2D, 2E). Telomerase is essential for maintaining telomere length, which is a critical parameter in the regulation of T cell expansion. We measured and compared telomerase and telomere length of stimulated naive T cells by these two modes of activation. We found MB-TCA tended to induce higher telomerase activity than D-TCA did (Supplemental Fig. 1A) and that MB-TCA–stimulated cells appeared to have an overall slower rate of decrease in telomere length than that of D-TCA for the duration of the cultures (Supplemental Fig. 1B). Similarly, the maintenance of telomere length of CD8^+^ T cells was better with MB-TCA than with D-TCA (Supplemental Fig. 1C). But none of these differences reached statistical significance analyzed by the LME model.

### Higher production of IL-10, IFN-γ, and TNF-α by D-TCA than by MB-TCA

**FIGURE 3.**
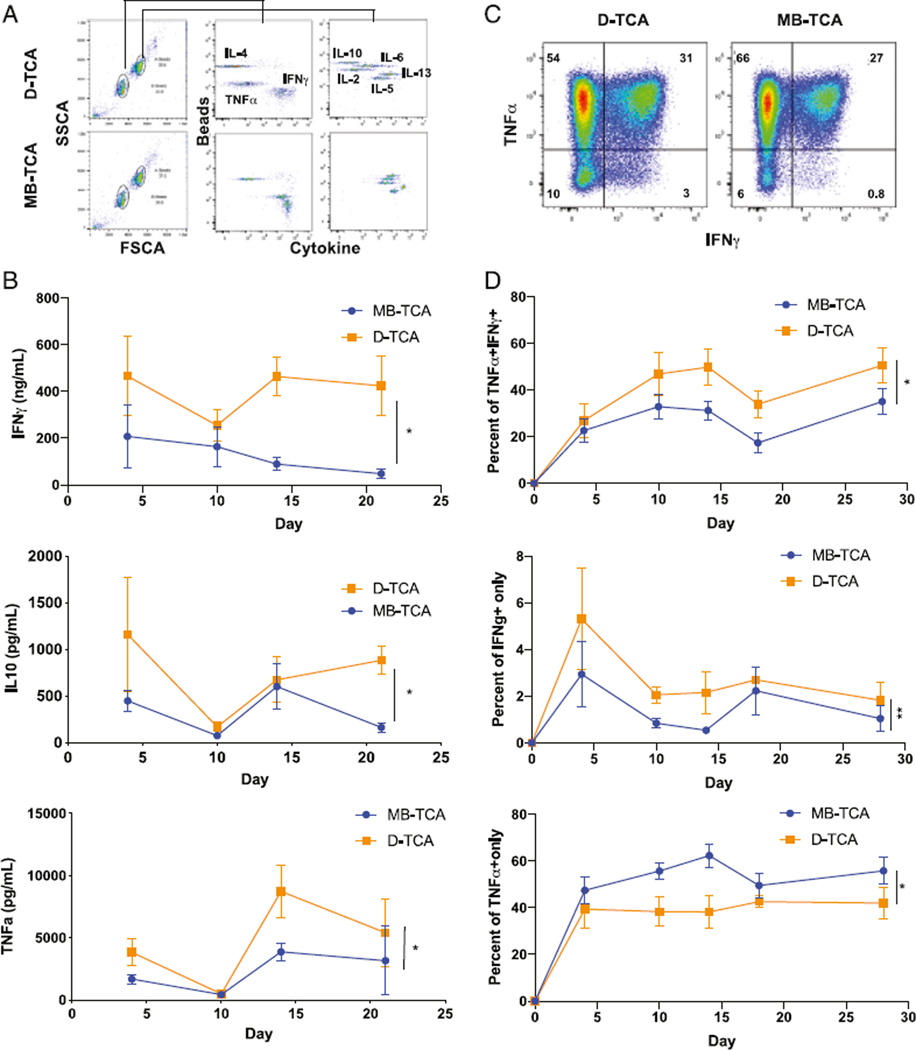
Cytokine levels in culture supernatants and inside of naive CD4^+^ T cells after stimulation with either D-TCA or MB-TCA. **(A)** Representative graph of cytokine levels by LEGENDplex method. A total of eight cytokines were measured as indicated. **(B)** Level of IFN-γ, IL-10, and TNF-α in culture supernatants of a 24-d course by either D-TCA or MB-TCA. Average values of 12 subjects are shown. **(C)** Representative graph of flow cytometry analysis of IFN-γ and TNF-α intracellular expression in stimulated naive CD4^+^ T cells. **(D)** Average percentages of cells producing both IFN-γ and TNF-α, or only IFN-g or TNF-a (*n* = 8). Comparison between D-TCA and MB-TCA was done by LME model. *p < 0.05, **p < 0.01.

Cytokines produced by stimulated T cells play a critical role in T cell growth and death. To determine whether cytokines were produced differently between MB-TCA and D-TCA, we measured eight common cytokines (INF-γ, TNF-α, IL-2, IL-4, IL-5, IL-6, IL-10, and IL-13) in the supernatant of stimulated naive CD4^+^ T cells at four time points: days 4, 10, 14, and 21 poststimulation using the LEGENDplex Assay Kit from BioLegend. Overall, we found that D-TCA–stimulated naive CD4^+^ T cells had significantly higher levels of IL-10, IFN-γ, and TNF-α than MB-TCA (Fig. 3A, 3B), but there were no significant differences in the other four cytokines (IL-2, IL-4, IL-5, IL-6, and IL-13) (Supplemental Fig. 3). We then measured intracellular levels of IFN-γ and TNF-α in stimulated naive CD4^+^ T cells by flow cytometry. Naive CD4^+^ T cells stimulated with D-TCA had a significantly higher percentage of TNF-α/IFN-γ double expressing T cells than MB-TCA did (Fig. 3C, 3D). Interestingly, D-TCA–stimulated naive CD4^+^ T cells also had a greater percentage of cells only expressing IFN-γ, whereas MB-TCA–stimulated naive CD4^+^ T cells had higher percentage of only expressing TNF-α (Fig. 3D).

### Blocking TNF-α improved naive CD4^+^ T cell expansion under D-TCA stimulation

**FIGURE 4.**
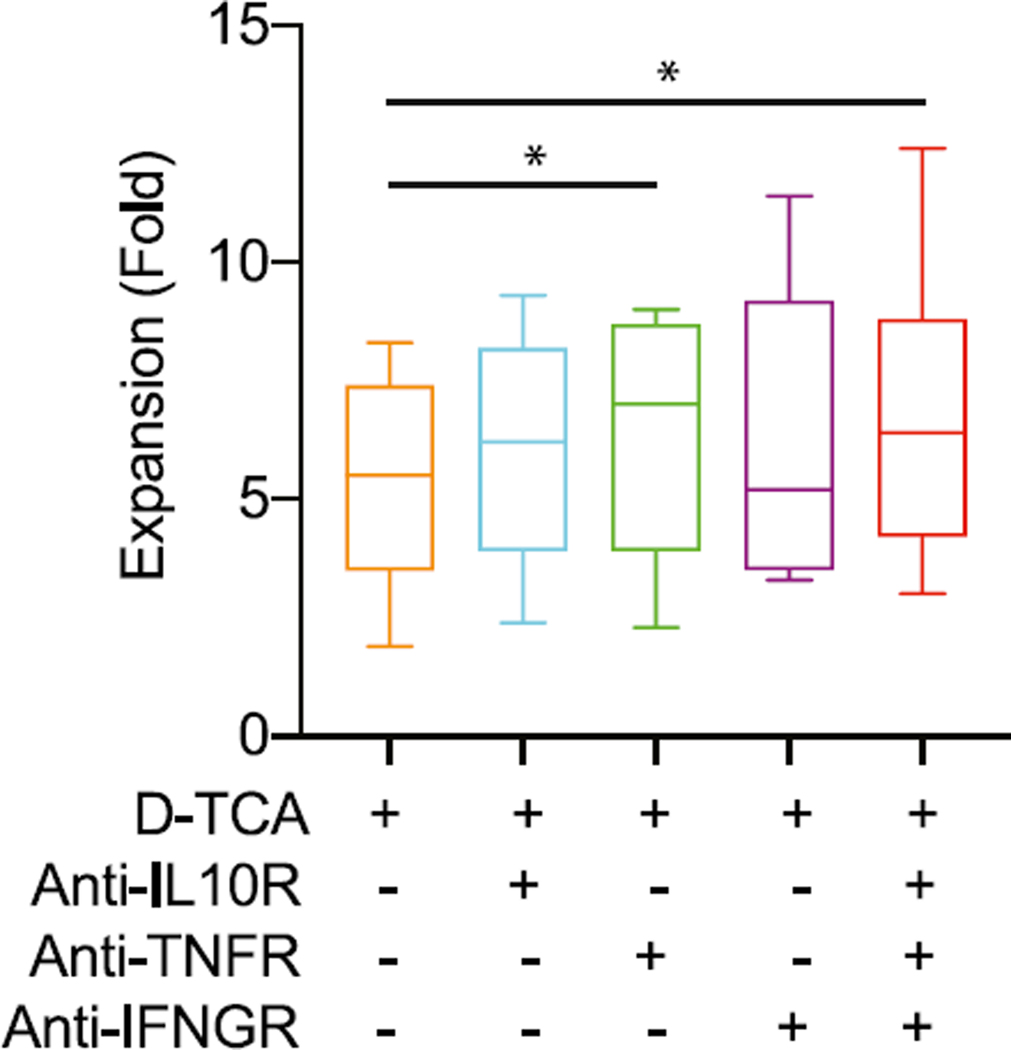
Effects of blocking cytokine receptors on growth of D-TCA–activated cells. One million naive CD4^+^ T cells were stimulated by D-TCA in the absence or presence of one Ab against one of the cytokine receptors (IFN-γ, IL-10, and TNF-α) or all three receptors. The cells were counted on day 7, and fold growth was calculated and presented (*n* = 7). Student t test was used for comparing blocking Ab and control. *p < 0.05.

To determine whether increased levels of IL-10, IFN-γ, or TNF-α contribute to reduced cell expansion under D-TCA, we applied cytokine receptor–specific blocking Abs against the three cytokines individually or in combination at the start of stimulation of naive CD4^+^ T cells and the cell expansion was measured at day 7 poststimulation. In the presence of individual Ab, blocking TNF-αR (TNF-αR1/TNFRSF1A) had significantly increased cell expansion but there was no significant effect from blocking receptors of IL-10 (IL-10Rα) or IFN-γ (IFN-γR1) alone (Fig. 4). The improved cell expansion was also observed under blocking of all three receptors simultaneously (Fig. 4). Together, these findings indicated increased TNF-α production under D-TCA was partially responsible for reduced naive CD4^+^ T cell expansion in vitro compared with MB-TCA.

### D-TCA reduced MB-TCA-activated cell expansion

**FIGURE 5.**
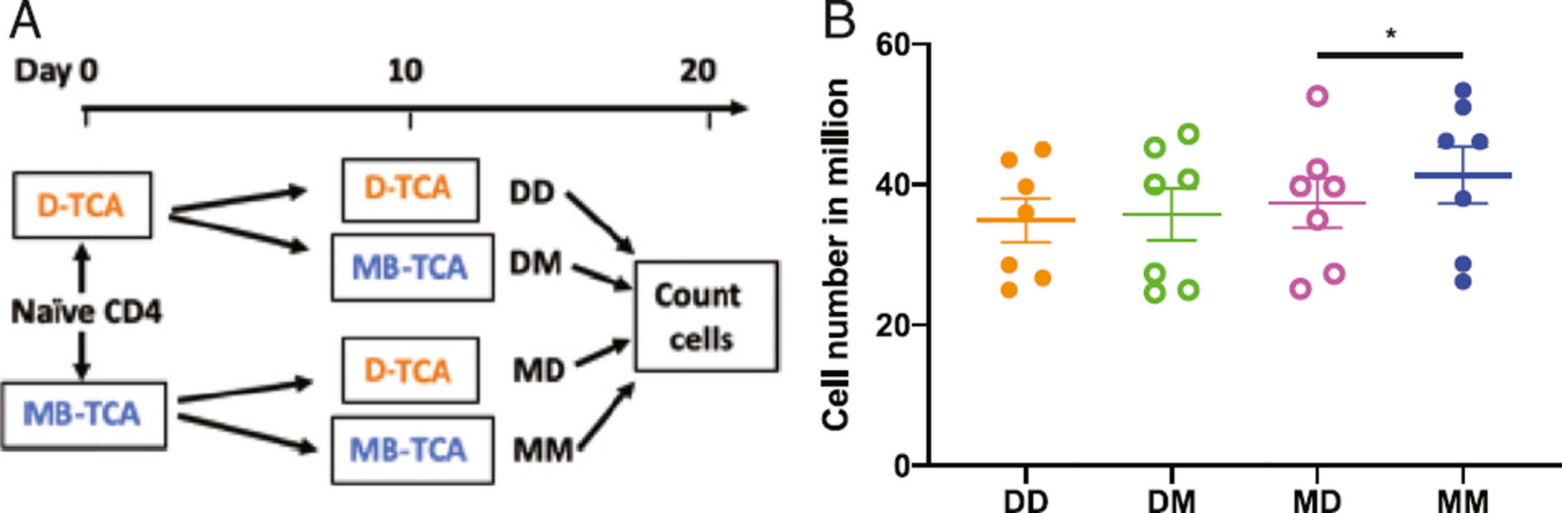
MB-TCA rescue of D-TCA–activated cultures. **(A)** Diagram of experiment scheme for variation of activators. Five million naive CD4^+^ T cells were stimulated by D-TCA or MB-TCA at day 0. The cells were counted on day 10, and 5 million stimulated cells from each culture were then reactivated with either D-TCA or MB-TCA again. The cells were then counted on day 20. **(B)** Number of cells at day 20 of culture under four different stimulation conditions (DD, DM, MD, and MM) are presented (*n* = 7). Student t test was used for comparing different conditions. *p < 0.05.

To determine whether the effects of MB-TCA or D-TCA on stimulated naïve CD4^+^T cells were reversible, we stimulated naive CD4^+^T cells with either MB-TCA or D-TCA for 10d and then split those cultures and restimulated with the same stimulator or the other stimulator for another 10 d. Cell numbers were counted in each of the four conditions and compared (Fig. 5A). We found that the proliferative response of naive CD4^+^ T cells that were originally stimulated with D-TCA was not improved when they were subsequently stimulated by MB-TCA (Fig. 5B). However, naive CD4^+^ T cells that were originally stimulated with MB-TCA had significantly poorer expansion when they were subsequently stimulated by D-TCA than by MB-TCA (Fig. 5B). Collectively, these findings suggest that D-TCA has potent impact on stimulated T cells, as both the initial and subsequent stimulations have a cumulative impact MB-TCA-induced expansion of stimulated naive CD4^+^ T cells.

## DISCUSSION

We have applied lipid MB-conjugated anti-CD3 and CD28 Abs (MB-TCA) as an activator for human naive T cells in vitro. Compared with the standard D-TCA, MB-TCA induces significantly better expansion of naive T cells in long-term culture. The superior expansion of T cells by MB-TCA over D-TCA is not due to faster cell divisions but rather better survival of activated T cells under repeated stimulations. Finally, naive T cells stimulated by MB-TCA produceless TNF-α than cells stimulated by D-TCA, and blocking TNF-α action via an Ab against TNF-αR1 (TNFRSF1A) significantly improved expansion of T cells activated by D-TCA in vitro. Thus, MB-TCA provides a superior stimulator to activate and expand large numbers of human T cells, which can be used for both basic and clinical applications.

Previous studies show that activation of human T cells induces telomerase activity, which, in turn, maintains telomere length of activated T cells undergoing robust division (22, 23). Maintenance of telomere length depends on the levels of induced telomerase in T cells, and reduced telomerase activity leads to rapid loss of telomere length and decreased expansion in vitro (24, 25). Interestingly, both MB-TCA and D-TCA tended to induce telomerase and maintain telomere length well in the early culture. However, in the later phase of culture, MB-TCA–activated T cells seemed to express higher levels of telomerase activity and longer telomere length than D-TCA T cells. Although it was not quite a statistically significant difference, this suggests that MB-TCA may provide an environment of activated T cells to better maintain telomerase expression and telomere length than D-TCA does in long-term cultures.

An optimal T cell activation and proliferation requires cytokine production. Because both MB-TCA and D-TCA shared anti-CD3 and CD28 Abs and induced similar initial human naive T cell expansion, we measured the levels of several cytokines in secreted form (culture supernatant) and within the stimulated cells and found that MB-TCA had consistently lower amounts of IL-10, IFN-γ, and TNF-α in the supernatants as well as lower percentages of IFN-γ^+^ only and TNF-α ^+/^IFN-γ^+^ double-positive cells than D-TCA. IFN-γ and TNF-α could induce both proliferation and apoptosis of activated T cells (15, 26) and IL-10, as a regulatory cytokine has also shown inhibitory effects on T cell proliferation (27, 28). We show in this study that blocking the receptor of TNF-αR1 (TNFSR1A), but not receptors of IL-10 (IL-10Rα) and/or IFN-γ (IFN-γR1), in D-TCA–stimulated naive CD4^+^ T cell cultures significantly improved expansion at day 7 of culture. Furthermore, blocking of all three cytokines also improved the cell expansion, but it is not substantially better than using anti–TNF-αR1 alone, suggesting that it is the greater production and/or signaling of TNF-α that appears to have a detrimental effect on the growth of D-TCA–stimulated cells.

Although MB-TCA is a better stimulator for long-term naive T cell expansion in vitro, it is not without some weakness. Because of the nature of the gas-filled lipid MB, its shelf life at 4°C ranges from weeks to months. Frequently mixing the MB-TCA at each use often leads to bursting of the MBs, which will require recount and adjustment of their concentration for each application so that the cell/MB ratio remains constant in each experiment. Improving the stability of MB-TCA will make the products more user friendly and require less frequent counting. Even with this weakness, MB-TCA offers an inexpensive stimulator for better expansion of human T cells in vitro, especially in long-term culture when the number of expanded T cells is preferred for both basic and clinical applications.

## Supplementary Material

Supplementary material

## Abbreviations used in this article:

7-AAD: 7–aminoactinomycin D
D-TCA: Dynabeads Human T-Activator
FSC-A: forward light scatter area
FSC-H: forward light scatter height
LME: linear mixed effects
MB: microbubble
MB-TCA: MB-based human T cell activator
MFI: mean fluorescence intensity
PFH: perfluorohexane
SSC-A: side light scatter area

## References

[1] Malissen B, Grégoire C, Malissen M, and Roncagalli R. 2014. Integrative biology of T cell activation. Nat. Immunol 15: 790–797. 25137453 10.1038/ni.2959

[2] Weng NP, Araki Y, and Subedi K. 2012. The molecular basis of thememory T cell response: differential gene expression and its epigenetic regulation. Nat. Rev. Immunol 12: 306–315. 22421787 10.1038/nri3173 PMC4686144

[3] Chang JT, Wherry EJ, and Goldrath AW. 2014. Molecular regulation of effector and memory T cell differentiation. Nat. Immunol 15: 1104–1115. 25396352 10.1038/ni.3031 PMC4386685

[4] Van Wauwe JP, De Mey JR, and Goossens JG. 1980. OKT3: amonoclonal anti-human T lymphocyte antibody with potent mitogenic properties. J. Immunol 124: 2708–2713. 6966296

[5] June CH, Ledbetter JA, Gillespie MM, Lindsten T, and Thompson CB. 1987. T-cell proliferation involving the CD28 pathway is associated with cyclosporine-resistant interleukin 2 gene expression. Mol. Cell. Biol 7: 4472–4481. 2830495 10.1128/mcb.7.12.4472-4481.1987 PMC368131

[6] Schwab R, Crow MK, Russo C, and Weksler ME. 1985. Requirements for T cell activation by OKT3 monoclonal antibody: role of modulation of T3 molecules and interleukin 1. J. Immunol 135:1714–1718. 3926880

[7] Sagerström CG, Kerr EM, Allison JP, and Davis MM. 1993. Activation and differentiation requirements of primary T cells in vitro. Proc. Natl. Acad. Sci. USA 90: 8987–8991. 8415642 10.1073/pnas.90.19.8987 PMC47486

[8] Levine BL, Ueda Y, Craighead N, Huang ML, and June CH.1995. CD28 ligands CD80 (B7–1) and CD86 (B7–2) induce long-term autocrine growth of CD4+ T cells and induce similar patterns of cytokine secretion in vitro. Int. Immunol 7: 891–904. 7577797 10.1093/intimm/7.6.891

[9] O’Connor RS, Hao X, Shen K, Bashour K, Akimova T, Hancock WW, Kam LC, and Milone MC. 2012. Substrate rigidity regulates human T cell activation and proliferation. J. Immunol 189: 1330–1339. 22732590 10.4049/jimmunol.1102757 PMC3401283

[10] Cheung AS, Zhang DKY, Koshy ST, and Mooney DJ. 2018. Scaffolds that mimic antigen-presenting cells enable ex vivo expansion of primary T cells. Nat. Biotechnol 36: 160–169. 29334370 10.1038/nbt.4047 PMC5801009

[11] June CH, O’Connor RS, Kawalekar OU, Ghassemi S, and Milone MC. 2018. CAR T cell immunotherapy for human cancer. Science 359: 1361–1365. 29567707 10.1126/science.aar6711

[12] Curtsinger JM, Schmidt CS, Mondino A, Lins DC, Kedl RM, Jenkins MK, and Mescher MF. 1999. Inflammatory cytokines provide a third signal for activation of naive CD4+ and CD8+ T cells. J. Immunol 162: 3256–3262. 10092777

[13] Thompson CB, Lindsten T, Ledbetter JA, Kunkel SL, Young HA, Emerson SG, Leiden JM, and June CH. 1989. CD28 activation pathway regulates the production of multiple T-cell-derived lymphokines/cytokines. Proc. Natl. Acad. Sci. USA 86: 1333–1337. 2465550 10.1073/pnas.86.4.1333 PMC286684

[14] Schafer PH, Wang L, Wadsworth SA, Davis JE, and Siekierka JJ. 1999. T cell activation signals up-regulate p38 mitogen-activated protein kinase activity and induce TNF-alpha production in a manner distinct from LPS activation of monocytes. J. Immunol 162: 659–668. 9916683

[15] Tateyama M, Oyaizu N, McCloskey TW, Than S, and Pahwa S.2000. CD4 T lymphocytes are primed to express Fas ligand by CD4 cross-linking and to contribute to CD8 T-cell apoptosis via Fas/FasL death signaling pathway. Blood 96: 195–202. 10891451

[16] Shi G, Cui W, Benchimol M, Liu YT, Mattrey RF, Mukthavaram R, Kesari S, Esener SC, and Simberg D. 2013. Isolation of rare tumor cells from blood cells with buoyant immuno-microbubbles. PLoS One 8: e58017. 23516425 10.1371/journal.pone.0058017 PMC3596333

[17] Shi G, Cui W, Mukthavaram R, Liu YT, and Simberg D. 2013. Binding and isolation of tumor cells in biological media with perfluorocarbon microbubbles. Methods 64: 102–107. 23974072 10.1016/j.ymeth.2013.08.008 PMC3841068

[18] Hess K, Yang Y, Golech S, Sharov A, Becker KG, and Weng NP.2004. Kinetic assessment of general gene expression changes during human naive CD4+ T cell activation. Int. Immunol 16: 1711–1721. 15492022 10.1093/intimm/dxh172

[19] Araki Y, Wang Z, Zang C, Wood III WH., Schones D, Cui K, Roh TY, Lhotsky B, Wersto RP, Peng W, 2009. Genome-wide analysis of histone methylation reveals chromatin state-based regulation of gene transcription and function of memory CD8+ T cells. Immunity 30: 912–925. 19523850 10.1016/j.immuni.2009.05.006 PMC2709841

[20] Rufer N, Dragowska W, Thornbury G, Roosnek E, and Lansdorp PM. 1998. Telomere length dynamics in human lymphocyte subpopulations measured by flow cytometry. Nat. Biotechnol 16: 743–747. 9702772 10.1038/nbt0898-743

[21] Hathcock KS, Hodes RJ, and Weng N-P. 2004. Analysis of telomere length and telomerase activity. Curr. Protoc. Immunol 62: 10.30.1-10.30.27. 10.1002/0471142735.im1030s62 18432920

[22] Weng NP, Levine BL, June CH, and Hodes RJ. 1996. Regulated expression of telomerase activity in human T lymphocyte development and activation. J. Exp. Med 183: 2471–2479. 8676067 10.1084/jem.183.6.2471 PMC2192611

[23] Liu K, Schoonmaker MM, Levine BL, June CH, Hodes RJ, and Weng NP. 1999. Constitutive and regulated expression of telomerase reverse transcriptase (hTERT) in human lymphocytes. Proc. Natl. Acad. Sci. USA 96: 5147–5152. 10220433 10.1073/pnas.96.9.5147 PMC21831

[24] Weng NP, Palmer LD, Levine BL, Lane HC, June CH, and Hodes RJ. 1997. Tales of tails: regulation of telomere length and telomerase activity during lymphocyte development, differentiation, activation, and aging. Immunol. Rev 160: 43–54. 9476664 10.1111/j.1600-065x.1997.tb01026.x

[25] Patrick MS, Cheng NL, Kim J, An J, Dong F, Yang Q, Zou I, andN. P. Weng. 2019. Human T cell differentiation negatively regulates telomerase expression resulting in reduced activation-induced proliferation and survival. Front. Immunol 10: 1993. 31497023 10.3389/fimmu.2019.01993 PMC6712505

[26] Church LD, Goodall JE, Rider DA, Bacon PA, and Young SP.2005. Persistent TNF-alpha exposure impairs store operated calcium influx in CD4+ T lymphocytes. FEBS Lett. 579: 1539–1544. 15733870 10.1016/j.febslet.2005.01.051

[27] Fernandez-Ponce C, Dominguez-Villar M, Aguado E, and Garcia-Cozar F. 2014. CD4+ primary T cells expressing HCV-core protein upregulate Foxp3 and IL-10, suppressing CD4 and CD8 T cells. PLoS One 9: e85191. 24465502 10.1371/journal.pone.0085191 PMC3896374

[28] Mazza G, Sabatos-Peyton CA, Protheroe RE, Herman A, Campbell JD, and Wraith DC. 2010. Isolation and characterization of human interleukin-10-secreting T cells from peripheral blood. Hum. Immunol 71: 225–234. 20034527 10.1016/j.humimm.2009.12.003 PMC3399767

