# Supplementary material for: Lipid Microbubble–Conjugated Anti-CD3 and Anti-CD28 Antibodies (Microbubble-Based Human T Cell Activator) Offer Superior Long-Term Expansion of Human Naive T Cells In Vitro
Source: Immunohorizons. Author manuscript; Available in PMC 2023 Aug 17. (PMC10433792; doi:10.4049/immunohorizons.2000056)
Supplement: Supplementary material [file NIHMS1918415-supplement-Supplementary_material.pdf]

Lipid microbubble conjugated anti-CD3 and anti-CD28 antibodies (MB-TCA) offer superior long-term expansion of human naïve T cells *in vitro*

Ana Lustig*, Ty'Keemi Manor*, Guixin Shi†, Jiangyuan Li*, Ying-Ting Wang†, Yang An‡, YuTsueng Liu§, and Nan-ping Weng*

* Laboratory of Molecular Biology and Immunology, National Institute on Aging, National

Institutes of Health, Baltimore,

Maryland, 21224 † Diagnologix LLC,

San Diego, CA 92121

‡ Laboratory of Molecular Biology and Immunology, National Institute on Aging, National Institutes of Health, Baltimore, Maryland, 21224

§ University of California at San Diego, CA 92093

Supplementary materials:

Supplementary Figure S1 Telomerase induction and telomere length maintenance, and growth and viability in MB-TCA and D-TCA stimulated naïve T cells

Supplementary Figure S2 Comparison of response to varying cell-to-bead ratio

Supplementary Figure S3 Cytokine measurements in culture supernatants

Supplementary Table S1 Donor information and used in the experiments

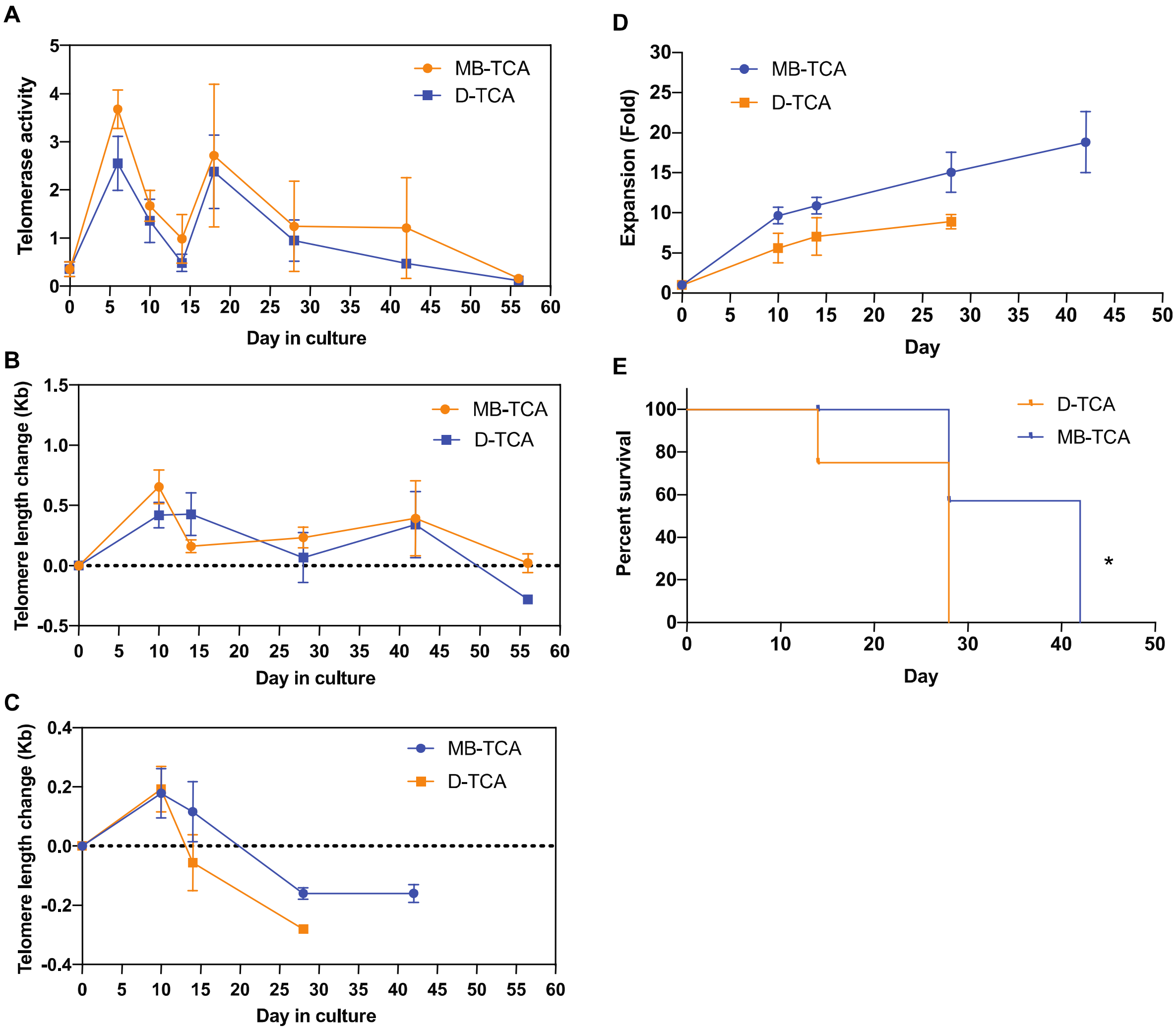

FIGURE S1. Telomerase induction and telomere length maintenance, and growth and viability of in MB-TCA and D-TCA stimulated naïve T cells. (A) Telomerase activity in stimulated naïve CD4+ T cells by MB-TCA or D-TCA. Telomerase activity was determined by the TRAP assay and relative telomerase activity was presented (Subject=13, average subject of each time point=6 with range 1-13). (B) Telomere length of stimulated naïve CD4+ T cells by MB-TCA or D-TCA. Telomere length was determined by Flow-FISH method and the average differences between day 0 and subsequent days (10, 14, 24, 28, 42, and 56) are presented in both D-TCA and MB-TCA stimulated cells. (subject=10, average of each time point=6 with range from 1-10). (C) Telomere length changes in D-TCA and MB-TCA stimulated naïve CD8+ T cells. n=7. (**D**) Cells were activated at day 0 with either D-TCA or MBTCA and re-stimulated every 14 days for the duration of the culture. At the designated time points cells were counted, and the fold growth of the culture was calculated as the total fold change in cell number from the beginning of the culture. Statistical analysis was done using the student's T test for paired samples. For the comparison of overall growth of the cultures at all time points, the n number was 14 paired comparisons from 6 independent experiments (p=0.019). For each time point, the paired n number is as follows: day 10= 6 pairs, day 14= 6 pairs, day 28= 2 pairs. Since so few cultures survived beyond day 28, we were unable to do paired statistics on those time points individually. (**E**) Longevity of naïve CD8+ T cells in culture stimulated by either D-TCA or MB-TCA. With naïve CD8+ T cells from a total of five subjects, three subjects stopped culture before day 28 when stimulated by D-TCA whereas no subject stopped before day 28 when stimulated by MBTCA. Gehan-Breslow-Wilcoxon test was used for comparison and p=0.02.

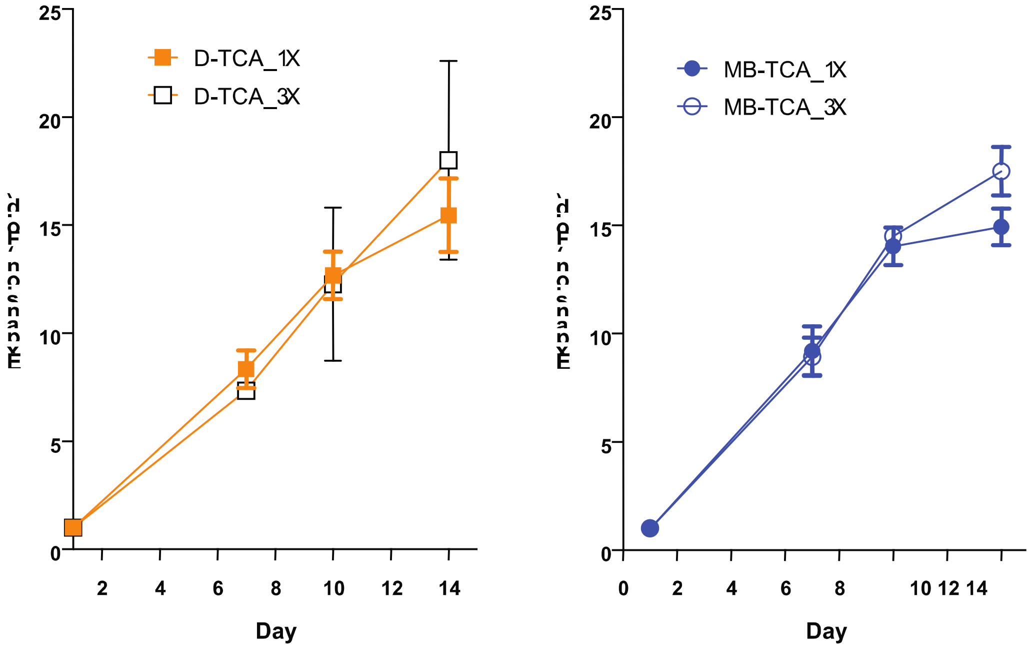

FIGURE S2. Comparison of response to varying cell-to-bead ratio. Naïve $CD4^{+}$ T cells were activated as described above with either a 1:1 cell:bead ratio as used for all experiments, or with a 1:3 ratio. Data is a result of 2 experiments with three cultures.

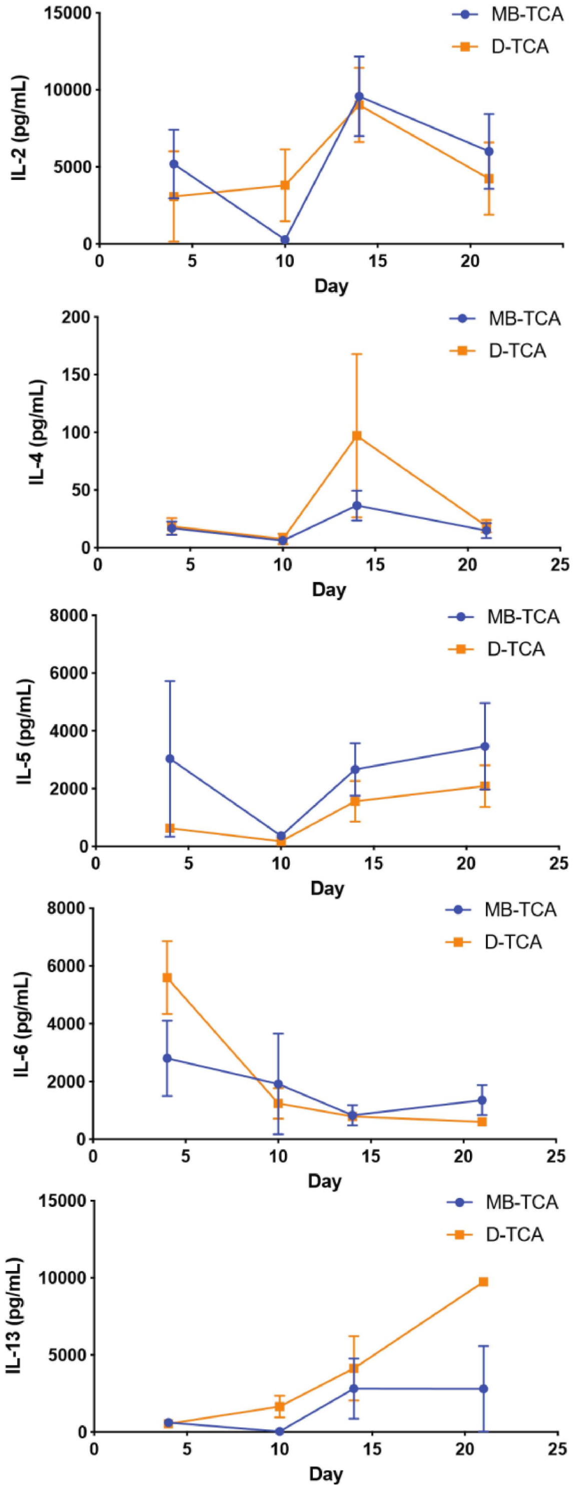

FIGURE S3. Cytokine levels in culture supernatants of naïve CD4+ T cells after stimulation with either D-TCA or MB-TCA. Graph of cytokine content in culture supernatants which did not appear to be statistically different between the two modes of activation (total of 25 experiments, analyzed in two batches).

Table S1 Donor information and used in the experiments*

Donor	Age	Gender	CD4-G	CD8-G	CD4-V	CD8-V	1Xv3X	CFSE	AnV	TL	Tel-ase	S-C	I-C	BL	DxM
1	53	M	X	X	X	X				X	X				
2	37	F	X	X	X	X				X	X				
3	51	M	X	X	X	X				X	X				
4	74	M	X	X	X	X				X	X				
5	38	M								X	X				
6	54	F								X	X				
7	39	F	X	x	x	x				X	X				
8	52	F	X	X	X	X				X	X				
9	53	M	X				X				X				
10	39	M	X	X	x	x					X				
11	50	M					X				X				
12	54	F	X		x						X				
13	42	M										X			x
14	37	M										X			
15	40	M										X			x
16	63	M								x	x				
17	64	M								X	X	x			x
18	39	F								X	X	X			X
19	51	M										X			X
20	55	F										X			X
21	40	M										X			X
22	39	F										X			X
23	47	F										X			X
24	47	F											X		
25	54	F						x					X		
26	65	M						X					X		
27	29	F						X					X		
28	64	M	X		x				X		X		X		
29	72	M	X		X			x	X		X		X		
30	47	F	X		X			X	X		X		X		
31	55	M	X		X			X	X		X		X		
32	73	M							X					X	
33	54	F							X					X	
34	55	M							X					X	
35	65	M							X					X	
36	40	F												X	
37	58	M												X	
38	53	M												X	
39	44	M												X	
40	31	M												X	
41	37	F												X	
42	66	M					X								
43	69	F					X								

* CD4-G and CD8-G refer to longterm culture of these cells. CD4-V and CD8-V refer to viability analysis. 1Xv3X refers to 1X and 3X concentration of D-TCA and MB-TCA experiment. AnV refers to Annexin V and 7-AAD experiment. TL=telomere length. Tel-ase=telomerase acitivity. S-C refers to cytokine measurement in supernatant, and I-C refers to intracellular staining of cytokine by flow cytometry. BL=Ab blocking experiment. DxM refers to D-TCA and MB-TCA switch experiment. X depicts data used from each donor for analysis.
